# Outcomes of Ultrasound-guided Glen Humeral Corticosteroid Injections in Adhesive Capsulitis

**DOI:** 10.9734/BJMMR/2015/13478

**Published:** 2014-09-25

**Authors:** Amos Song, Jeffrey N. Katz, Laurence D. Higgins, Joel Newman, Andreas Gomoll, Nitin B. Jain

**Affiliations:** 1 Department of Orthopaedic Surgery, Brigham and Women’s Hospital and Harvard Medical School, Boston, Massachusetts, United States; 2 Division of Graduate Medical Sciences, Boston University School of Medicine, Boston, Massachusetts, United States; 3 Division of Rheumatology, Immunology, and Allergy, Brigham and Women’s Hospital and Harvard Medical School, Boston, Massachusetts, United States; 4 Department of Radiology, New England Baptist Hospital, Boston, Massachusetts, United States; 5 Department of Physical Medicine and Rehabilitation, Spaulding Rehabilitation Hospital and Harvard Medical School, Boston, Massachusetts, United States; 6 Harvard Shoulder Service, Harvard Medical School, Boston, Massachusetts, United States; 7 Departments of Physical Medicine and Rehabilitation and Orthopaedics, Vanderbilt University Medical Center, United States

**Keywords:** Ultrasound, corticosteroids, adhesive capsulitis, injections

## Abstract

**Aims:**

To assess short and longer-term outcomes of ultrasound-guided glenohumeral corticosteroid injections for adhesive capsulitis.

**Study Design:**

A mixed prospective and retrospective study design

**Place and Duration of Study:**

Department of Physical Medicine and Rehabilitation, Spaulding Rehabilitation Hospital, Department of Orthopaedic Surgery, Brigham and Women’s Hospital, between June 2011 and July 2012.

**Methodology:**

Using medical records, we first retrospectively identified patients who had received ultrasound-guided injections of lidocaine and triamcinolone for adhesive capsulitis We then assessed short-term follow-up outcomes (within 3 months of procedure) using medical record review and phone interviews. Longer-term follow-up (at least 3 months from the procedure) outcomes were determined by mailings and phone calls. Average and worst shoulder pain scores were measured on a visual analog scale. Shoulder ROM was measured in forward flexion, isolated abduction, and external rotation.

**Results:**

Patients presented an average of 5.1 (SD=4.1) months after onset of symptoms. Within three months of the injection, 55.9% (95% CI: 39.2%, 72.6%) of patients reported greater than 75% pain relief and 44.1% (95% CI: 27.4%, 60.8%) of patients reported greater than 75% ROM improvement. The percentage of patients who improved increased with increased duration of follow-up. At short-term follow-up (mean=2.1 months, SD=2.7), average pain decreased from 5.6 (SD=2.2) to 3.0 (SD=1.8) (p ≤ .001) and worst pain decreased from 7.8 (SD=1.2) to 4.3 (SD=3.2) (p ≤ .001). At longer-term follow-up (mean =10.4 months, SD=3.7), average pain decreased to 1.9 (SD=1.9) (p ≤ .001) and worst pain decreased to 2.9 (SD=2.3) (p ≤ .001).

**Conclusion:**

A majority of patients had significant pain reduction and functional improvement after an ultrasound guided glenohumeral corticosteroid injection for adhesive capsulitis. Our patients experience the majority of their pain and functional relief within the first three months after an ultrasound-guided corticosteroid injection with continued increase in relief in the longer-term.

## 1. INTRODUCTION

Adhesive capsulitis is a debilitating shoulder condition of unknown etiology. In addition to pain, there is a significant loss of active and passive range of motion. Adhesive capsulitis most commonly affects women of ages 40–60 [1,2]. Diabetes [3,4], hyperthyroidism [5], hypothyroidism [6], and hypertension [1] are associated with adhesive capsulitis. Although adhesive capsulitis is a self-limited condition, symptoms can last from one to two years [7] and residual symptoms may linger even longer [8,9].

Treatment options for adhesive capsulitis include non-steroidal anti-inflammatory drugs, physical therapy, manipulation under anesthesia, dilation or distension of the shoulder joint, arthroscopic capsular release, and oral or intra-articular corticosteroids. The joint capsule is the site of pathology in adhesive capsulitis and glenohumeral corticosteroid injections target this location. Glenohumeral injections performed without image-guidance can be inaccurate and fail to be consistently placed in the glenohumeral joint [10–13]. Hence, imaging by ultrasound can be used to visually guide the injection of corticosteroids into the glenohumeral joint. However, few studies have examined the short and longer-term outcomes of ultrasound-guided glenohumeral corticosteroid injections for adhesive capsulitis [14,15].

We assessed short-term and longer-term effects of ultrasound-guided glenohumeral corticosteroid injections on shoulder pain and range of motion. We also assessed factors associated with better outcomes after an ultrasound-guided corticosteroid injection for adhesive capsulitis.

## 2. METHODOLOGY

### 2.1 Patient Population

Ethics approval for this study was obtained from the Institutional Review Board at our institution.

Electronic billing records were used to retrospectively identify patients who had received an ultrasound-guided, intra-articular corticosteroid injection for adhesive capsulitis from June 1, 2011 to December 31, 2012. One of three shoulder/sports fellowship-trained physicians (NJ, LH, or AG) made the diagnosis of adhesive capsulitis based on presence of shoulder pain and limitation in active and passive range of motion, and shoulder x-rays to rule out osteoarthritis.

There were 67 potential subjects ascertained from our billing records. Patients were excluded if they were either non-English speaking (n=4) or did not present for follow-up within 3 months of the injection (n= 12). Four patients received a second injection for reasons such as a shoulder trauma since the first injection or because of incomplete relief from the first injection. These patients were excluded to avoid bias in our results. No patients reported major complications due to an injection.

Our final patient population included 47 patients/51 shoulders (Table 1). Of these, all 47 patients were evaluated at short-term follow up and 36 patients at longer-term follow-up. The mean age of the included patients was 57.9 (SD=9.1, range: 34, 79) years (Table 1). Female patients comprised 70.2% of the study population. The percentage of patients self-identifying as white was 76.6%. The average duration of symptoms before the first visit was 5.1 months (SD=4.2, range: 0.5, 15).

### 2.2 Ultrasound Guided Corticosteroid Injections

**Fig. 1:**
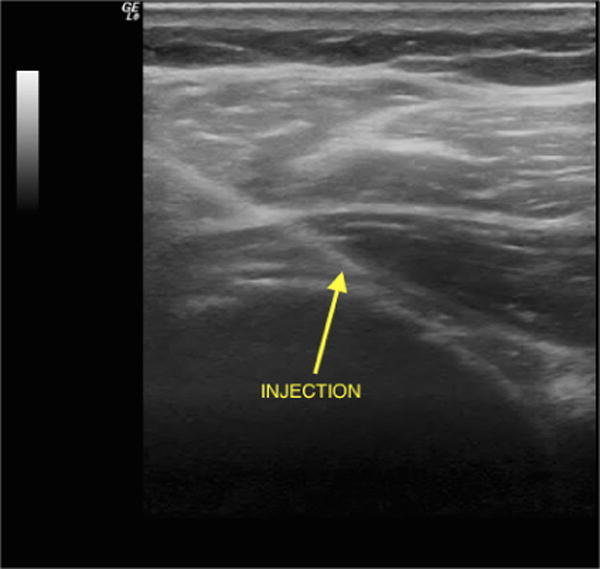
An ultrasound showing the needle approaching the glenohumeral joint

All injections were performed by a single physician (NJ). Patients were injected using a posterior, lateral to medial, approach while seated or in lateral decubitus. Ultrasound guidance was performed using a 5.5 megahertz curved linear array or a 13–8 megahertz linear transducer. Fig. 1 shows an ultrasound image of the lateral to medial posterior approach injection into the glenohumeral joint.

A 20, 21, or 22 gauge spinal needle was used to inject approximately 4 milliliters of 2% or 1% lidocaine, and 40 milligrams of triamcinolone acetonide (Kenalog, Bristol-Myers Squibb Company, Princeton, NJ, USA) into the glenohumeral joint. After the injection, patients were referred to physical therapy or asked to begin a home exercise program in 2–4 weeks so as to allow for pain relief prior to initiation of stretching exercises that are otherwise painful in adhesive capsulitis.

### 2.3 Outcome Measures

Patients reported average and worst pain during 2 weeks prior to the injection using the visual analog scale (VAS) of 0 to 10. Subjective shoulder value (SSV) 16 was assessed on a scale of 0 to 100, where 100 represented a completely normal shoulder that has no pain and is fully functional and 0 represented one that has the worst possible pain and function. Range of motion was measured in forward flexion, isolated abduction, and external rotation in neutral by the attending physician in all cases (NJ).

### 2.4 Short-Term and Longer-Term Follow-Up

Patients were followed up at two time points – within 3 months of the injection (short-term) and at greater than 3 months since the injection (longer-term). Patients were routinely scheduled for a follow-up visit in clinic within 3 months (n=35) when they completed an injection questionnaire that included average and worst pain scores and SSV. ROM was measured by the attending physician at this visit. Pain scores, SSV, and ROM measures were then retrospectively retrieved from electronic medical records. As part of this study protocol, patients not yet due for an in-clinic follow-up visit at the start of the study (i.e. less than 3 months after the injection) were called by phone to assess pain scores and SSV (n=12).

**Fig. 2:**
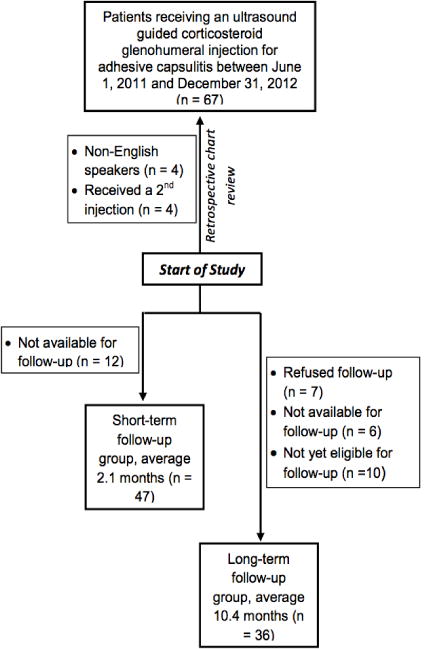
Flow diagram of patient inclusion into short-term and longer-term follow-up groups

Longer-term follow-up was defined as a follow-up conducted 3 or more months after the corticosteroid injection. A total of 36 patients available for longer-term follow-up were administered a telephone questionnaire where they were asked a structured series of seven questions pertaining to the pain and function of their affected shoulder(s) that included average and worst pain scores and SSV. The questionnaire also assessed the length of time to obtain >75% pain relief and >75% range of motion improvement (range of motion was not measured objectively by the patient or provider in longer-term follow-up). Fig. 2 shows a timeline of patient inclusion into short-term and longer-term follow-up groups.

### 2.5 Statistical Analysis

Basic descriptive statistics were performed with means and standard deviations for continuous variables and number and percent for categorical variables. Race/ethnicity was defined as a dichotomous variable – white and non-white. Paired t-tests were used to compare continuous variables (change in average pain and worst pain, change in percent of average pain and worst pain, and change in forward flexion, isolated abduction, and external rotation) between paired groups of observations. Linear regression was used to identify the association between changes in shoulder outcome measures such as average pain, worst pain, and ROM and factors such as age, gender, race, duration of pain in months, and presence of diabetes, hyperthyroidism or hypothyroidism, or hypertension. Patient characteristics used for regression variables were selected based on prior studies showing possible associations with adhesive capsulitis. Statistical analysis was performed using Microsoft Excel 2007 for Windows.

## 3. RESULTS AND DISCUSSION

### 3.1 Results

The mean short-term follow-up duration was 2.1 (SD=2.7) months. Significant improvements were found in pain scores for average pain and worst pain, range of motion measurements for forward flexion and isolated abduction in patients with short-term follow-up. Average pain decreased from 5.6 (SD=2.2) to 3.0 (SD=1.8) (p ≤ .001) and worst pain decreased from 7.8 (SD=1.2) to 4.3 (SD=3.2) (p ≤ .001). Forward flexion increased from 96.6 (SD=29.4) degrees to 128.2 (SD=19.6) degrees (p ≤ .001) and isolated abduction increased from 52.3 (SD=16.5) degrees to 83.0 (SD=16.1) degrees (p ≤ .001) (Table 2).

Improvements in subjective shoulder value and external rotation did not reach statistical significance in short-term follow-up. The mean longer-term follow-up duration was 10.4 (SD=3.7) months. There were significant improvements in subjective shoulder value, average pain, and worst pain as compared to their baseline values in longer-term follow-up. Average pain decreased to 1.9 (SD=1.9, p ≤ .001), worst pain decreased to 2.9 (SD=2.4, p ≤ .001), and subjective shoulder value increased to 73.3 (SD=22.7, p =.02).

**Fig. 3:**
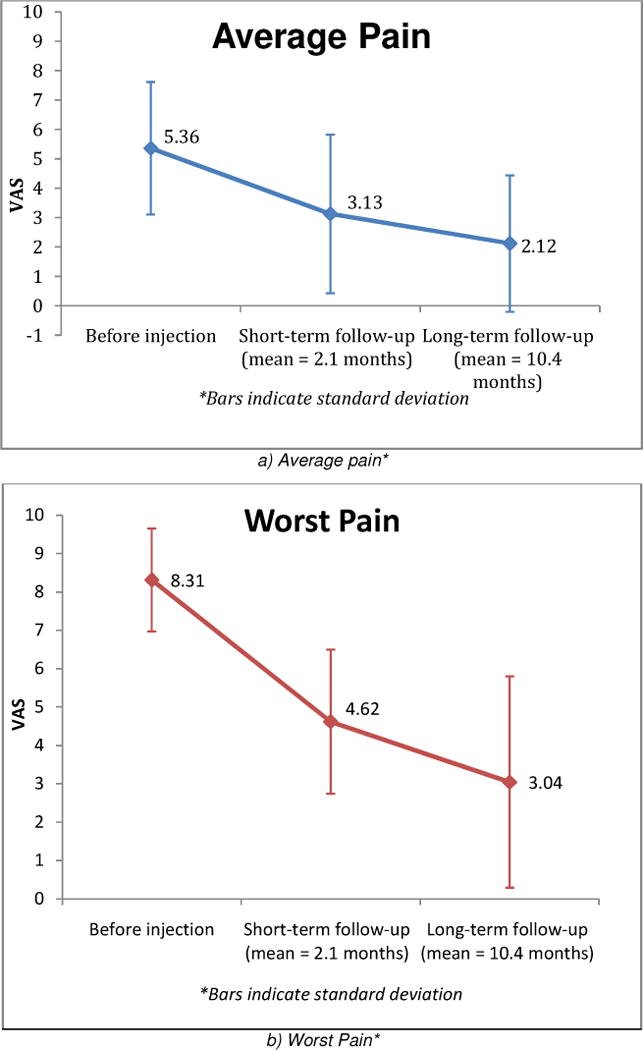
Pain improvement in patients with adhesive capsulitis undergoing ultrasound guided glenohumeral injection

Unlike Table 2, Fig. 3 only includes patients who completed both short-term and longer-term follow up (n = 36 patients, 40 shoulders). Average pain decreased from 5.36 to 3.13 at short-term follow-up (mean = 2.1 months) and to 2.12 at long-term follow-up (mean = 10.4 months). Worst pain decreased from 8.31 to 4.62 at short-term follow-up and to 3.04 at long-term follow-up.

**Fig. 4:**
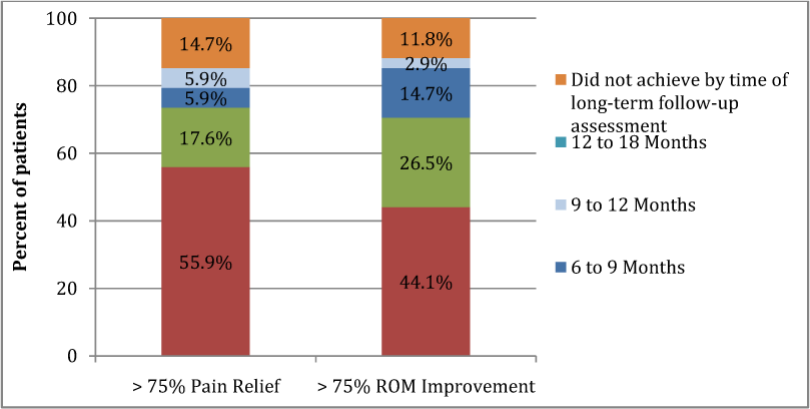
Time to >75% improvement in pain and range of motion in patients with adhesive capsulitis undergoing ultrasound guided corticosteroid injection

A majority of patients (55.9%; 95% CI=39.2–72.6%) achieved greater than 75% pain relief in the first three months after the injection and 44.1% (95% CI=27.4–60.8%) of patients self-reported greater than 75% range of motion relief within three months of the injection (Fig. 4). Within six months, 73.5% of patients reported greater than 75% pain relief and 70.6% of patients reported greater than 75% improvement in range of motion. Patients also reported an average of 80.0% overall improvement in shoulder pain and an 81.8% overall improvement in shoulder function from baseline at longer-term follow-up.

Age, sex, diabetes/thyroid disease, and hypertension were not significantly independently associated with either short-term or longer-term outcomes of shoulder injections for adhesive capsulitis. There was a weak association between patients who self identified as white and improvements in longer-term pain outcomes. Patients identifying as white had an additional 31.1% improvement of average pain, additional 24.2% improvement in worst pain, and an overall 2.7 points greater improvement in worst pain score. This association was not observed for any of the short-term outcome measures. There was also a weak, but significant, association between duration of pain and change in external rotation (β = −0.004; p=.045) in short-term follow-up.

### 3.2 Discussion

We assessed short-term and longer-term outcomes of patients undergoing glenohumeral injections under ultrasound guidance for adhesive capsulitis. The results of this study indicate that patients with adhesive capsulitis generally experience rapid improvement in shoulder pain and range of motion outcomes after an ultrasound guided corticosteroid injection. Most patients experience improvements in pain and range of motion within 3 months of the injection. Further, smaller improvements are seen at longer-term evaluation.

Ultrasound-guided injections accurately deliver corticosteroids into the glenohumeral joint [10–14,17]. Only a few prior studies have assessed image-guided corticosteroid injections for adhesive capsulitis by either ultrasound or fluoroscopy. These studies reported rapid pain and motion relief [14, 15, 18–23]. Carette et al. found that fluoroscopically guided corticosteroid injection groups had significantly higher improvements in Shoulder Pain and Disability Index (SPADI) scores after 6 weeks compared to physical therapy or placebo[23]. Lorbach et al. found significant improvements in American Shoulder and Elbow Shoulder (ASES) score, SF-36, and ROM scores beginning 4 weeks after a fluoroscopically guided corticosteroid injection [15]. Lee et al. found significantly greater ROM and pain improvements in an ultrasound guided injection group compared to an unassisted injection group by the second week but not beyond the third week [14]. Although image-guided corticosteroid injections can rapidly improve shoulder outcomes in the treatment of adhesive capsulitis, there is still not a general consensus in the literature on the overall effectiveness of these injections in the longer-term, particularly after 12 weeks. Our data show that patients experience significant pain and function relief in the short-term follow-up (average 2.1 months) that is maintained, and improved, in longer-term follow-up (average 10.4 months). However, we acknowledge that our study did not have a control group. Hence, we cannot ascertain whether patients might have improved over several months without an injection. It should be noted though, that patients in our study presented on an average of 5.1 months after onset of symptoms without spontaneous relief.

Some randomized trials have shown that corticosteroid injections offer no significant improvement in pain improvement as compared with a control group by the end of their study periods [18–20, 22–30]. Although all of these studies claimed their injections were intra-articular, more than half of these trials did not utilize image-guided shoulder injections[24–30]. Glenohumeral injections performed without image-guidance can be inaccurate and fail to consistently reach their intended target [10–13]. Sethi et al. found only 26.8% of unassisted injections intended for the glenohumeral joint were actually intra-articular [10]. Hence, prior randomized trials may be biased towards the null due to inaccurate placement of corticosteroid in the glenohumeral joint. Our study design did not allow for a placebo controlled group or a group that underwent subacromial injections as a control group to assess this hypothesis. Future studies in this area are needed.

To the best of our knowledge, there are no studies investigating the factors associated with better outcomes after corticosteroid injections for adhesive capsulitis. Our analysis of determinants of better outcomes in short-term and longer-term follow-up after glenohumeral injections was exploratory given the relatively small sample size. A longer duration of symptoms prior to injection was associated with a smaller change in external rotation range of motion during short-term follow-up. A possible explanation for this finding is that patients may already be in the second (freezing) phase of adhesive capsulitis if their presentation was delayed after the onset of symptoms. Hence, better range of motion improvement may potentially be achieved if the injection is performed earlier after onset of symptoms. Patients who self-identified as white also had better outcomes on some but not all measures. This finding needs to be replicated in larger studies to make definitive conclusions.

Limitations of our study include the absence of a comparison group, such as a placebo group or a physical therapy only group because our study was not a randomized controlled trial. No patients in our study received unguided injections because ultrasound guidance is considered the best injection practice at our clinic. In addition, the limited sample size precluded identification of factors with modest effects on outcome. Also, the potential effect of other co-interventions such as physical therapy may have an impact on longer-term outcomes.

## 4. CONCLUSION

In conclusion, patients experience significant improvements in shoulder function and pain after an ultrasound-guided, intra-articular corticosteroid injection for adhesive capsulitis, with most of the improvement occurring early on in the treatment timeline. Patients also report continued improvement in shoulder function and pain in the longer-term. Further investigation is needed to determine the predictors of better outcomes.

## Figures and Tables

**Table 1:** Baseline characteristics of the 47 included patients (51 shoulders)

Patient characteristics	
Mean age (SD)	57.9 (9.1)
Male	29.8%
Female	70.2%
**Affected side(s)**	
Left shoulder	46.8%
Right shoulder	44.7%
Bilateral cases	8.5%
Duration of symptoms in months (SD)	5.1 (4.2)
**Comorbidities**	
Diabetes	10.6%
Hyperthyrodism / hypothyroidism	17.0%
Hypertension	19.1%
**Race/ethnicity**	
% Black, Hispanic, Asian, or Unknown	23.4%
% White	76.6%

SD=Standard deviation

**Table 2:** Short-term and longer-term outcomes of ultrasound guided glenohumeral injections

Outcome measure	Before injection, (Mean ± SD)	Follow-up, (Mean ± SD)	Two tail p-value
**Short-term outcomes**^1^			
Subjective Shoulder Value (SSV)	54.0 (24.1)	64.0 (19.5)	.1
Average pain	5.6 (2.2)	3.0 (1.8)	≤ .001
Worst pain	7.8 (1.2)	4.3 (3.2)	≤ .001
Forward flexion (FF)	96.6 (29.4)	128.2 (19.6)	≤ .001
Isolated abduction (IA)	52.3 (16.5)	83.0 (16.1)	≤ .001
External rotation (ER)	40.0 (17.6)	43.3 (21.1)	.09
**Longer-term outcomes**^2^			
Subjective Shoulder Value (SSV)	48.8 (21.4)	73.3 (22.7)	.02
Average pain	5.2 (2.2)	1.9 (1.9)	≤ .001
Worst pain	8.4 (1.5)	2.9 (2.4)	≤ .001

1 mean duration = 2.1 (2.7) months, (n = 51),

2 mean duration = 10.4 (3.7) months, (n = 40)
